# Discretionary Fortification—A Public Health Perspective

**DOI:** 10.3390/nu6104421

**Published:** 2014-10-17

**Authors:** Tarasuk Valerie

**Affiliations:** Department of Nutritional Sciences, Faculty of Medicine, University of Toronto, Toronto, ON M5S 3E2, Canada; E-Mail: valerie.tarasuk@utoronto.ca; Tel.: +1-416-978-0618; Fax: +1-416-978-5882

**Keywords:** discretionary fortification, novel beverages, nutrient adequacy, population health, consumer information, food safety

## Abstract

‘Discretionary fortification’ refers to the addition of vitamins and minerals to foods at the discretion of manufacturers for marketing purposes, but not as part of a planned public health intervention. While the nutrients added may correspond to needs in the population, an examination of novel beverages sold in Toronto supermarkets revealed added nutrients for which there is little or no evidence of inadequacy in the population. This is consistent with the variable effects of manufacturer-driven fortification on nutrient adequacy observed in the US. Nutrient intakes in excess of Tolerable Upper Intake Levels are now observed in the context of supplement use and high levels of consumption of fortified foods. Expanding discretionary fortification can only increase nutrient exposures, but any health risks associated with chronically high nutrient loads from fortification and supplementation remain to be discovered. Regulatory bodies are focused on the establishment of safe levels of nutrient addition, but their estimation procedures are fraught with untested assumptions and data limitations. The task of determining the benefits of discretionary fortification is being left to consumers, but the nutrition information available to them is insufficient to allow for differentiation of potentially beneficial fortification from gratuitous nutrient additions.

## 1. Introduction

Food fortification has long been employed as a strategy to address demonstrated problems of nutrient insufficiency in populations. Although the specific regulatory mechanisms governing such fortification differ across jurisdictions, the guiding principles established by the Codex Alimentarius Commission [1] have served as an international standard for the addition of essential nutrients to foods. ‘Discretionary fortification’ marks a significant departure from these principles. Sometimes termed ‘liberal’ or ‘voluntary’ fortification, it is the addition of vitamins and minerals at the discretion of food manufacturers for marketing purposes, but not as part of a planned public health intervention. Discretionary fortification is thus distinct from voluntary fortification programs implemented by public health authorities to address specific population health problems (e.g., the introduction of voluntary folate fortification in Australia and New Zealand in the 1990s as a strategy to reduce the risk of neural tube defects [2]). In countries such as the United States, where most fortification is voluntary, discretionary fortification denotes nutrient additions that fall outside established standards of identity, nutrition quality guidelines, and other relevant regulations [3]. In countries like Canada, where nutrient additions aligned with Codex principles are tightly regulated, discretionary fortification simply describes fortification activity outside mandatory programs. The nutrients added through discretionary fortification may correspond to nutrient needs in the population, but the defining feature of this fortification practice is that it occurs outside any defined public health strategy.

It is argued that discretionary fortification can provide consumers with a greater variety of sources for nutrients and thus help them to meet their requirements [4,5]. However, concerns have been expressed that expanding fortification may function in opposition to the promotion of healthy eating [6], and that it may expose populations to risks of nutrient toxicity. Fortification has been associated with intakes of some nutrients above tolerable upper intake levels (ULs) [7,8]. Whether such intake levels constitute serious threats to health is a subject of ongoing debate [4,9], but the findings highlight the potential for excessive intakes in the context of uncontrolled additions of micronutrients to the food supply. Regulators are thus being challenged to determine safe levels of nutrient addition and establish procedures to monitor the effects of discretionary fortification on the population.

As the practice of discretionary fortification unfolds, it is important to consider the public health implications of this phenomenon. In this paper, some distinguishing features of discretionary fortification are illustrated, drawing on data from a recent study of novel beverages sold in Toronto supermarkets. Emerging literature from the U.S. and elsewhere is then reviewed to consider the population health implications of discretionary fortification. Finally, the particular challenges that discretionarily fortified foods pose to regulators and the implications of this practice for consumers are discussed.

## 2. Discretionary Fortification: A Case Study of ‘Novel’ Beverages Sold in Toronto Supermarkets

In 2010–2011, we conducted a comprehensive survey of front-of-package nutrition-related marketing of all packaged foods and beverages in three supermarkets in Toronto, representing the three largest grocery chains in Canada [10,11]. The survey captured 20,520 unique products. Discretionarily fortified beverages were identified by the presence of “Natural Health Product” on their principal display panel as this designation denoted product approval via a regulatory framework (the Natural Health Products Directorate) that, at the time of the study, permitted manufacturer-driven nutrient additions to food products. Eighty such beverages were identified, and upon return to the stores, 66 were found and purchased for analysis. The nutrient content of these beverages was compared to Dietary Reference Intakes and a content analysis of the product labels was conducted [12].

Our sample of ‘novel’ beverages included 33 caffeinated energy drinks, 19 enhanced waters, and 14 novel juices. Eighty-five percent of products were manufactured or distributed by Coca-Cola or PepsiCo, highlighting the global nature of the products. The micronutrient content of the beverages per stated serving size is summarized in Table 1. The discretionary fortification practices exemplified in these products are distinct from more controlled fortification designed to address defined public health problems in several ways:

i) Only some of the nutrients added are ones for which there is a palpable prevalence of inadequacy among young adults in the Canadian population [13], yet they appear to be the target market for the products. While young adults could benefit from the added vitamin A, potassium, vitamin D, calcium, and magnesium found in some products, the most commonly added nutrients were B-vitamins for which there is little or no evidence of inadequate intakes among this population subgroup. The estimated prevalence of inadequacy for vitamins B-6 and B-12 is higher among older adults (peaking at 32.5% among Canadian women over 70 years of age [13]), but the text and imagery on the products examined do not suggest that they are targeted to this age group.
ii) Several beverages were fortified with nutrients for which no population dietary assessment has been conducted–specifically pantothenic acid, vitamin E, or chromium. Thus, it is impossible for food manufacturers, regulators, or consumers to gauge how the products relate to current intake levels or needs in the population.
iii) In contrast to the carefully controlled nutrient additions associated with public health interventions, the levels of addition in these beverages varied widely. In many cases, a single serving furnished much more than the human requirement for a nutrient. When compared to the requirement estimates for young adult men (*i.e.*, the age-sex group with the highest requirements for the nutrients reported, and thus the most conservative comparator), 18 beverages contained more than six times the Estimated Average Intake (EAR) for vitamin B12, 25 contained more than triple the EAR for vitamin B6, 13 contained more than three times the EAR for niacin, 14 contained more than three times the EAR for riboflavin, and seven contained more than four times the Adequate Intake for pantothenic acid.
iv) Whereas the calibration of nutrient additions to the energy content or gram weight of particular food vehicles is critical to managing exposure when fortification is implemented as a public health measure, the energy content of products in this study ranged from 0 to 230 kcal/serving (mean = 113 kcal), and low- and zero-calorie products were as likely as more energy dense ones to contain nutrients in excess of requirements. The fortification of calorie-free beverages that might be regarded as alternatives to water (*i.e.*, ‘vitamin waters’) suggests the potential for consumption of multiple servings, heightening nutrient loads.

## 3. Appraising the Population Health Impact of This Practice 

The merits of nutrient additions to specific food products can be evaluated relative to the nutritional needs of target market groups, but assessing the population health impact of discretionary fortification as a policy direction requires analysis of the effects of this practice on usual nutrient intakes in the population. Much of the literature in this area is speculative, including studies that have modeled the impact of specific policy proposals [14,15,16,17] or proposed maximum safe levels of addition [18] on estimated distributions of usual intake in the population. While consistently identifying the potential for the expansion of discretionary fortification to result in intake distributions over ULs, such research is limited by the need to impose assumptions about food manufacturers’ uptake of the opportunities afforded by changing regulatory environments and consumers’ responses to product innovations. Further insight can be derived from research into the current effects of food fortification on usual nutrient intakes in countries where discretionary fortification is practiced.

**Table 1:** Nutrient content of ‘novel’ beverages per serving in relation to nutrient requirements and estimated prevalence of inadequacy for Canadian men, 19–30 years.

Nutrient	Number of Beverages (*n* = 66) *n* (%)	Median Content/Serving (Minimum–Maximum)	Estimated Average Requirement	*n* (%) > Estimated Average Requirement	Canadian Population Prevalence of Inadequacy ^4^
Vitamin A (mcg)	13 (20) ^1^	375 (17–3000)	625	6 (9)	47.4%
Vitamin B6 (mg)	50 (76)	3.5 (0.1–8.7)	1.1	31 (47)	<5%
Vitamin B12 (mcg)	42 (64)	5.5 (1.0–26)	2.0	34 (52)	<5%
Vitamin C (mg)	34 (52)	137 (15-205)	75	20 (30)	13.7%
Vitamin D (mcg)	2 (3)	1.7 ^2^	10.0	0	78%
Vitamin E (mg)	20 (30)	7.8 (2.0–31)	12.0	3 (5)	NA
Folic acid (mcg)	3 (5)	100 (100–200)	320	0	<5%
Niacin (mg)	47 (71)	20 (0.6–50)	12.0	28 (42)	<5%
Pantothenic acid (mg)	31 (47)	5.3 (1.6–25)	5.0^3^	20 (30)	NA
Riboflavin (mg)	23 (35)	3.4 (0.1–7.0)	1.1	22 (33)	<5%
Thiamin (mg)	1 (2)	0.1	1.0	0	<5%
Calcium (mg)	9 (14)	100 (2.2–570)	800	0	25.4%
Chromium (mcg)	4 (6)	41 ^2^	35 ^3^	4 (6)	NA
Magnesium (mg)	3 (5)	36 (27–40)	330	0	34.8%
Potassium (mg)	3 (5)	350 (319–400)	4700 ^3^	0	86.2% < AI
Zinc (mg)	4 (6)	3.8 (1.8–3.8)	9.4	0	<5%

^1^ Includes 10 beverages containing retinol palmitate and 3 containing beta-carotene; ^2^ All products had the same amount; ^3^ An Estimated Average Requirement has not been determined, so this value represents the Adequate Intake; ^4^ Estimated from the Canadian Community Health Survey, 2004, based on intakes from food alone. Values are presented as ‘<5%’ because the extreme sampling variability at the lower end of the distribution precludes reliable estimation of more exact estimates. ‘NA’ indicates that dietary intake data are not available for this nutrient. Table adapted from Dachner *et al*. [12].

### 3.1. Improved Nutrient Intakes

The US presents a particularly fertile ground for studies of discretionary fortification because of its long history of manufacturer-driven fortification and extensive monitoring of dietary intakes. On any day, about half of the population consumes some discretionarily fortified food, typically breakfast cereals or beverages [8]. Drawing on data from the 2003–2006 National Health and Nutrition Examination Survey (NHANES), Fulgoni *et al.* established that enrichment and fortification had a substantial effect of the prevalence of inadequacy for vitamin A, thiamin, iron, and folate among both children and adults [7]. Fortification and enrichment also contributed substantially to intakes of niacin, riboflavin, and vitamins B6, B12, C, and D, but with less marked effects on nutrient adequacy. For many B-vitamins, the population prevalence of inadequacy was low even without added nutrients from fortification [7]. For other nutrients such as calcium, magnesium, and vitamins C and E, fortification appeared insufficient to compensate for suboptimal dietary intakes. Analyses of the contribution of fortified foods to usual nutrient intakes in other countries also indicate that their consumption diminishes the prevalence of inadequacies for some but not all nutrients, and some additions appear unnecessary [5,19,20,21]. These results highlight the haphazard nature of the effects of manufacturer-driven fortification; benefits only accrue when the nutrients added are not already attained in sufficient amounts from natural sources and when the scale of fortification is sufficient to shift otherwise inadequate intakes to adequate levels. Neither condition is assured when fortification occurs at the discretion of manufacturers.

Throughout the foregoing discussion, benefit has been defined as usual nutrient intakes sufficient to meet current requirement estimates. It could be argued that some benefit might accrue from higher levels of intake, but the requirement estimates applied in the analyses summarized here represent intake levels associated with optimal health and reduced risk of chronic degenerative diseases, insofar as there was evidence to support the determination of these values [22]. Contrary to the standards used to set nutrient requirements in the past, the requirement estimates established through the Institute of Medicine’s Dietary Reference Intakes process exceed the nutrient intake levels required to avoid nutrient deficiencies. Reviews of new and emerging literature might reveal health benefits with higher intake levels for some nutrients, but our current requirement estimates constitute the most scientifically defensible estimates of nutrient needs for optimal health presently.

### 3.2. Risks of Excess

Analyses of the effects of fortification on the upper tails of estimated usual intake distributions suggest that in some instances, this practice may lead to nutrient exposures in excess of established ULs. Among 2–18 year olds in the 2003–2006 US NHANES, small proportions of the estimated distributions of usual intake for vitamin A, niacin, and folate surpassed the ULs when nutrient intakes from fortification and enrichment were taken into account [7]. Even without considering fortification, 10% of children’s zinc intakes exceeded the UL, but this rose to 18% when fortified sources were included [7]. When age groups were disaggregated further, 45% of 2–8 year olds were found to have usual zinc intakes (from natural and added sources) above the UL [23]. This analysis revealed no indication of intakes above the ULs for US adults [7], which is similar to findings from earlier examinations of the effects of voluntary fortification on the nutrient intakes of adults in Ireland [19] or Austria [24]. While these results have been interpreted to indicate that there is likely little or no risk of excessive nutrient exposures from discretionary fortification, given the marketing element of this practice, it is important to consider the possibility of differential nutrient exposure with different consumer practices.

Inter-individual variation in nutrient exposure is commonly observed with mandatory fortification because of between-person differences in food selection and consumption patterns, but the potential for such variation is magnified with discretionary fortification because by design this practice expands the array of food choices by introducing options with greater nutrient density. In an effort to differentiate consumption patterns within the US population, Sacco *et al.* estimated age- and sex-specific distributions of usual intake for quintiles defined by the probability to consume nutrients from voluntarily fortified foods, using data from the 2007–2008 NHANES [8]. Manufacturer-driven fortification was identified from food code descriptions, added nutrients, and ingredient lists, excluding foods with a standard of identity for enrichment or fortification. Among adults, increased consumption of calcium and iron from voluntarily fortified foods was associated with greater risk of intakes above the UL for some age/sex groups. Among children, higher intakes of zinc, retinol, folic acid, selenium, and copper from voluntarily fortified foods was associated with greater likelihood of intakes above the UL, with marked effects for some groups. For example, among 4–8 year olds, the proportion of usual zinc intakes above the UL ranged from 4.9% in the lowest quintile to 35.9% in the highest [8]. More research is required to identify the socio-demographic and behavioral characteristics of those most likely to consume high levels of discretionarily fortified foods.

Although supplement use was not factored into Sacco *et al.*’s analysis of nutrient intakes, they found that across several age/sex groups, individuals with higher intakes of voluntarily fortified foods were more likely to be supplement users [8]. This association implies increased potential for elevated nutrient loads among supplement users with the expansion of discretionary fortification. When nutrient intakes from both food and supplements have been considered, estimated distributions of usual intakes for all age/sex groups in Canada and the US have been found to surpass the ULs for a broad array of nutrients [7,23,25,26,27,28]. Dietary assessment studies in Europe and the United Kingdom have also documented nutrient exposures in excess of upper levels when supplement use is taken into account [21,29]. For the most part, the proportion of the population overall with intakes above ULs is under 10%, but these estimates apply to the entire population and thus understate the effect of supplements on the nutrient exposures of consumers. When a stratified analysis was undertaken with Canadian population data, considerably higher rates of intakes above the ULs were observed among some age/sex groups of supplement users for calcium, iron, zinc, magnesium, niacin, folic acid, and vitamin A [26]. Similarly, analyses of folic acid intake among U.S. adults and children stratified by nutrient source have documented higher likelihood of intakes above the UL in conjunction with the consumption of higher-dose supplements [27,28]. Among adults, the highest prevalence of folic acid intakes above the UL (12.8%) was among adults 60 years of age and older who consumed enriched grain products, ready-to-eat cereals with folic acid, and folate supplements [27]. Such intake patterns comprise the foundation of nutrient exposure upon which any expansion of discretionary fortification is being layered.

The ULs are the only available benchmark against which to appraise the safety of nutrient additions, but it is important to recognize their limitations. Defined as the highest average daily intake that is likely to pose no risk of adverse health effects to almost all individuals in the general population, the UL represents a ‘best estimate’ of a safe upper bound for a nutrient [30]. However, this is a nascent field in nutrition, and there is a paucity of data upon which to base determinations for many nutrients [31]. Thus, we have only crude estimates of safe upper ranges of intake for some nutrients and no estimates whatsoever for others [32]. Probability assessment methods cannot be applied to determine the prevalence of excessive intakes using the existing thresholds because the shape of the dose-response curve for adverse events is uncharted for most nutrients [33]. Several of the ULs that have been established differ across jurisdictions, reflecting different interpretations of the existing evidence [34]. In many instances, the ULs established for children have been extrapolated from data on adults, raising questions about the validity of these estimates [32]. However, the most important limitation of the ULs with respect to their application to set policy on discretionary fortification, elaborated by Rasmussen *et al.*, is the fact that the ULs we have now were established to evaluate the safety of current intake levels, not the safety of future fortification [31]. The health implications of long-term exposure to elevated doses of multiple vitamins and minerals from supplements and fortificants are unknown [31].

### 3.3. Influence on Dietary Patterns

Separate from questions about the safety of discretionarily fortified foods, there are concerns about the potential for this practice to promote or reinforce food consumption patterns that are deleterious to health [35,36]. Given regulatory frameworks that exclude staple foods from discretionary fortification (e.g., through standards of identity), the foods most likely to be targeted are processed foods with little intrinsic nutritional value. The fortification of energy dense, nutrient-poor foods that are widely consumed can effectively increase nutrient intakes, as illustrated, for example, by the contribution of vitamin C-fortified fruit drinks to US children’s total intakes [23]. Whether the benefits associated with improved micronutrient intakes outweigh the risks associated with the macronutrient profile of the food vehicle is a matter of debate, as is the question of whether the fortification of foods with undesirable nutritional attributes such as added sugars functions to foster greater consumption of such products. However, the case of fortified fruit drinks highlights the tension between discretionary fortification practices and public health strategies designed to promote dietary practices that support healthy body weights and lessen risks of diet-related chronic diseases. With foods high in added sugars now targeted for reduction in an effort to combat rising rates of obesity, public health measures that limit the consumption of sugar-sweetened fruit drinks in the US could adversely affect children’s micronutrient intakes [23].

## 4. Regulating Discretionary Fortification

### 4.1. Setting Safety Standards

As discretionary fortification practices expand, recognition of the potential risks of uncontrolled nutrient additions to the food supply is driving the development of safety standards. These may include prohibiting the addition of some nutrients (e.g., retinol) or delineating the nutrients allowed to be added at the discretion of manufacturers and setting limits on the amounts of particular nutrients that can be added per 100 kcal or portion of food. The mathematical modeling proposed to develop such standards juxtaposes the 95th percentile of the distribution of baseline nutrient intakes in the population (with or without consideration of nutrient exposures from supplement use) with Tolerable Upper Intake Levels [31,37]. Defining safe amounts for discretionary fortification then reduces to apportioning what Verkaik-Kloosterman and colleagues have referred to as the ‘free space’ between these two values [38]. Depending on how high the assumed UL for a nutrient is relative to current intake levels, these modeling procedures can easily yield safe levels of addition that are well in excess of human requirements, which perhaps explains the high levels of nutrient addition observed in our sample of ‘novel’ beverages. In one of the first published examples of such modeling, Flynn *et al.*, concluded that several nutrients, including riboflavin, pantothenic acid, and niacin (added as nicotinamide), could be safely added per 100 kcal portion in amounts several times the European Commission Recommended Daily Intake [37]. More sophisticated modeling approaches, taking into account safe levels of exposure estimated for children [31] and inter-individual variation in nutrient exposures from fortificants and supplements [18], have resulted in lower estimated thresholds for safe nutrient additions, highlighting the importance of considering intra-population variation in nutrient exposure and tolerance when setting safety standards. The results of these studies also highlight the sensitivity of safety standard estimation procedures to inter-jurisdictional differences in food selection practices and supplement use.

The myriad of assumptions and data limitations underpinning the estimation procedures being used to develop safety standards begs the question of how well regulators will be able to manage the potential risks associated with expanding discretionary fortification. As noted above, the benchmarks being applied to define upper safe levels of intake derive from very crude estimation procedures, in many cases with very limited data on toxicity; the resulting estimates are not necessarily indicative of safety in relation to the chronically high nutrient exposures that will result from expanded fortification and continued supplement use. The 95th percentile of usual nutrient intakes is also likely to be estimated with error. Above and beyond the biases in population survey data associated with underreporting, the nutrient contributions of fortified foods are very likely to be underestimated. The manufacturing practice of overage means that nutrient levels in foods consumed may be higher than those listed in food composition databases [31,39]. Furthermore, discretionarily fortified foods are not well captured in existing food composition databases and dietary intake surveys [39,40], because this is such a rapidly evolving practice. Errors in the assessment of nutrient intakes from fortified foods have serious implications for safety calculations premised on filling the ‘free space’ because fortified food consumers sit at the upper tails of population intake distributions; underestimation of their intakes means an overestimation of the potential for expanded fortification.

### 4.2. Managing Exposure through Labeling Regulations

In addition to restricting nutrient additions, regulatory bodies can influence food manufacturing practices with respect to fortification through the implementation of labeling regulations that function as marketing incentives and disincentives. In the U.S., for example, voluntary food fortification has been managed in part through the regulation of nutrient content claims that are tied to specific compositional criteria. It is assumed that manufacturers will fortify products to the levels required to enable them to make specific nutrient content claims (e.g., ‘product X is a good/excellent source of nutrient Y’), but there will be no marketing advantage to nutrient additions beyond the thresholds for these claims. The extent to which voluntary fortification practices are intertwined with nutrition labeling regulations in the U.S. is evident in the public health concerns spawned by plans to update the Daily Values used in nutrition labeling (*i.e.*, a reference standard based on recommended nutrient intakes) to reflect more current science [41]. If lower Daily Values are set for some nutrients, this could result in lower nutrient additions to widely consumed products because manufacturers need to add less to qualify for nutrient content claims, with potentially deleterious effects on nutrient adequacy in the population [3,41]. Further evidence of the dynamic relationship between regulated labeling and product formulation in the US can be found in the Institute of Medicine’s recent decision to not include an assessment of micronutrients in its recommendation for standardized front-of-package nutrition labelling, so as not to encourage more voluntary food fortification and increase the risk of excessive nutrient intakes [42].

As discretionary fortification expands, food manufacturers appear to be seizing opportunities for product innovation and taking new approaches to food marketing. While our examination of novel beverages was very limited in scale, the on-package marketing of these products was noteworthy. The presence of specific nutrients was typically indicated on the front-of-package, but most manufacturers eschewed regulated nutrient content claims, diet-related health claims, or other conventional nutrition references [12]. This occurred even though the levels of nutrient addition far exceeded thresholds required to make such claims. The novel beverages we examined were instead promoted on package labels through more ambiguous assertions about mental alertness, hydration, the replenishment of needed nutrients, and other social and psychological benefits that were less clearly related to their nutrient content [12]. Whereas the nutrient content of some discretionarily fortified foods could be expected to shift if there are changes to the Daily Values [41], our findings suggest that such changes will have no impact on the nutrient content of many novel beverages. This implies that more direct regulatory action is required for governments to manage discretionary fortification.

## 5. Discerning Benefit—The Consumer’s Job

The development of regulations based on the principle of filling the ‘free space’ without exceeding the upper bounds of safe intakes denoted by the current ULs could conceivably protect populations from excessive nutrient exposures, assuming estimates of exposure were accurate and the science underpinning whatever ULs were applied was sound and relevant to emerging fortification scenarios. Even with these caveats though, it is important to recognize that such regulatory action does not imply, let alone ensure, that discretionarily fortified foods will confer any benefit to those who purchase them. The task of discerning benefit in the context of market-driven food fortification is left entirely to the consumer. This extraordinarily difficult task is ill-supported by the nutrition information currently provided to consumers.

While the labels of discretionarily fortified foods may include nutrition and health claims, such text appears at the discretion of the manufacturer and it is part of product marketing. The only mandatory, standardized nutrition information on product labels in most jurisdictions is a Nutrition Facts table. (Until recently in Canada, even this table was not required on novel beverages because they fell under Natural Health Products regulations [43].) The table provides information on the nutrient contribution of one serving for selected nutrients, expressed as a percent of the Daily Value. Yet, it is not uncommon for novel beverages to report nutrient levels that are several hundred percent of the Daily Value. The on-package marketing of many beverages we examined included assertions that the products would ‘replenish’ or ‘restore’ missing nutrients [12], but the information required for consumers to appraise such assertions goes well beyond what is currently available. The Nutrition Facts table communicates nothing about the probability that a consumer stands to benefit, or could be placed at risk, from the nutrients listed. An extraordinarily high level of nutrition knowledge is required for a consumer to understand that he already has a very high probability of meeting his riboflavin or niacin requirement, for example, and thus is unlikely to benefit from consuming a beverage offering 500% of the Daily Value for that nutrient. Consumers cannot possibly differentiate between nutrients added that would help them to meet their requirements and nutrient additions that are simply about manufacturers filling the ‘free space’. Nutrition education programs could perhaps be developed to help consumers evaluate the potential risks and benefits of specific discretionarily-added nutrients, but this would necessitate training people to micro-manage their micronutrient loads.

## 6. Conclusions

Discretionary fortification represents a marked departure from food fortification designed to address public health needs. The potential for health benefits from manufacturer-driven fortification appears to be remote, given how common it now is for manufacturers to add nutrients with no evidence of need. Unfortunately, we are ill-equipped to appraise the risks of permitting expanded food fortification for marketing purposes. While there is a strong foundation of nutritional science upon which to determine the benefits (or lack thereof) of discretionary nutrient additions, the long-term health implications of chronic exposure to the high nutrient loads achievable through expanding fortification and supplement use are largely unknown. Thus, the risks of discretionary fortification remain to be discovered. As regulators now attempt to manage the safety of rapidly evolving fortification practices through crude estimates of the ‘free space’ left to be filled with nutrient additions, while leaving consumers to discriminate between possibly beneficial fortification and gratuitous nutrient additions, discretionary fortification is a policy direction in need of serious review.
